# An exploratory study on the publication stages of early access articles in different bibliographic databases: A case study of IEEE journals

**DOI:** 10.1371/journal.pone.0325787

**Published:** 2025-06-11

**Authors:** Yunu Zhu

**Affiliations:** Library of Zhejiang University, Hangzhou, People’s Republic of China; European Commission, ITALY

## Abstract

Currently bibliographic databases have included a large number of Early Access (EA) articles. Taking 47 IEEE journals as examples, this study analyzed and compared the differences in publication stages of EA articles in three typical bibliographic databases, including Web of Science Core Collection, Scopus, and Engineering Village Compendex. Qualitative analysis of data sets that may appear in these three databases and their publication stage modes, and quantitative analysis on the number of records, proportion, and journal distributions of each data set and each publication stage mode were conducted. There were totally 7 sub-data sets and corresponding 26 publication stage modes, with 14 “undifferentiated publication stage modes” and 12 “differentiated publication stage modes”. Although the proportion of EA records from each “differentiated publication stage mode” was mostly below 1.0%, the absolute quantity of EA records with differences in the publication stage was noteworthy reaching 2516. Among the 47 journals, 23 journals have 7–8 publication stage modes, 1 journal having 18 modes, and 40 journals have one or more “differentiated publication stage modes”. Therefore, in IEEE journals, whether for the same EA article or the same journal, the difference in publication stage between these three databases was pervasive and complex.

## 1 Introduction

Early Access (EA) is a common model in the academic publishing industry, derived from dual format (online and print) journals. This means that the article can be obtained online before it is officially published in a print journal. This model may play an important role in promoting the exchange of academic findings. Studies have shown that the availability of EA articles reduced publication delays [1–3], increased reader access [4–5], and provided researchers with the latest E-publications for systematic reviews [6]. In recent years, several popular bibliographic databases, such as Web of Science, Scopus, and Engineering Village, have collaborated with publishers to index a large number of EA records. According to rough search and statistics, as of January 4, 2024, the Web of Science Core Collection (WoS CC) has included 492,091 EA records (2017–2023), Scopus included 415,508 records (2011–2023), and Engineering Village Compendex (EI) included 164,188 records (2006–2023). It can be seen that in addition to journal websites or publishing platforms, bibliographic databases, as an important tool for literature retrieval and acquisition, have become another way for researchers to access EA articles. In addition, it is worth noting that the increasing number of EA records in bibliographic databases makes it possible to calculate new bibliometric indicators and research evaluation indicators. As announced by the Web of Science, EA content will be introduced in the calculation of journal impact factors starting from 2021 [7]. Therefore, EA articles not only change the basic data of the database, but also have an impact on research achievement acquisition, bibliometric analysis, and research performance evaluation.

In bibliographic databases, the indexing method of EA articles is different from traditional indexing methods. Traditionally, database only involves officially published articles (which have been assigned volume, issue, and page numbers), and does not include EA articles. For EA articles, database indexing is usually carried out in two stages. The first stage involves indexing the article during its early access phase, while the second stage involves cataloging it after its official publication. That is to say, EA articles indexed by the database will have two publication stages in sequence – early access stage and official publication stage. In addition, there are differences in the indexing methods for EA articles among different databases. The WoS CC retains the early access date when indexing the official publication stage of the EA article, while Scopus and EI delete completely the records of the early access stage after indexing the official publication stage. However, the introduction of EA articles and specific indexing methods may cause changes in database functionality and even some errors. In the Web of Science database, the “year published” field in search page can search for the online and final publication years of indexed records, while the “publication year” in the search results page only represents the online publication year [8]. Nowadays, WoS CC has added the function key of final publication year to the search results page. In the Scopus database, the retrieval results for Year of Publication and Date of Publication are inconsistent and one possible reason is that the two fields use different versions of the publication date [9]. In Scopus, after indexing the official version of an article, the link to citation obtained by the Online-First version may be lost [10–11]. Due to the different indexing policies of WoS CC and Scopus for publication dates, there may be large discrepancies in the number of records in the same journal included in these two databases [12]. These previous studies undoubtedly remind us that bibliographic databases may produce some problems in the process of EA article indexing. However, they did not involve a specific analysis the publication stage of EA articles in databases.

As the number of EA articles and their additional data in various databases continues to increase, it is necessary and urgent to conduct an evaluation accordingly. For EA articles, the dual publication stage is a unique mark that distinguishes them from pure electronic journal articles and pure print journal articles. It's closely related to the publication time of the article, which is a finer grained level of information than the article level. For bibliographic databases, the unique method of indexing the two publication stages separately makes the publication stage of EA articles a new feature field in the database, which is one of the parameters that may be involved in literature retrieval, literature analysis, and bibliometric work. Today, EA article indexing has become normal in databases. Just like other field information such as affiliation [13–18], funding [19–22], Digital Object Identifier (DOI) [23–26], author [27–29], document type [30–34], and language [32,35], we have to consider potential impact of publication stage in the evaluation, selection and application of databases. If the publication stage of the same EA article is different in different databases, that is, the publication stage information of the same EA article obtained through different databases is different, this will undoubtedly cause confusion, and it is impossible to determine which database is correct. Further, if different EA articles in the same journal have different publication stage modes, which will undoubtedly add to the confusion. To this end, this paper intends to conduct an exploratory study on the publication stage of EA articles and their differences in different bibliographic databases, in order to reveal the current situation and problems of EA article indexing in databases, especially the publication stage indexing. At present, there is little research specifically on the practice of indexing EA articles in bibliographic databases, and there is limited information to refer to. This paper intends to make a comprehensive and systematic analysis of the indexing characteristics and differences of EA article publication stage in common and typical bibliographic databases (including WoS CC, Scopus and EI) by combining qualitative and quantitative methods and examples. It is hoped that the research results can truly reflect the status and problems of publication stage indexing of EA articles in different databases, and provide valuable reference information for the development and use of databases.

## 2 Literature review

### 2.1 Research on the publication stage of EA articles

The publication stage of EA articles, especially the role of online publication and the delay between print publication and online publication, has sparked a series of studies. A survey on over 200 journals from different publishers showed that printed versions of journals were typically published an average of 3 months later than online versions [2]. The “advanced online publication” (AOP) mechanism in biomedical journals and food research journals reduced publication delays for papers [1,3]. The delay between print and online publication of neuroscience journals can artificially raise a journal's impact factor [36]. A 2010 survey found that advance online publication increased the impact factor of ophthalmic journals [37]. Online publication of in press articles affected severely the calculation of immediacy index of journals [38]. The publishing time (the period between submission and online availability) was negatively and significantly related to journal impact factor [39]. The impact factor and the immediacy index values of information science journals with early view mechanism were higher than the others [40]. Mendeley readers accrued and continued to steadily increase when articles were first available online [4]. Journals with “articles in press” mechanism had the citation advantage and higher CiteCore value [41]. The articles-in-press mechanism in energy and fuels journals was not significantly related to publication lag, but affected citation speed [42]. The electronic access increased after the articles’ online release and print publication [5].

Other research issues were the ways publishers and databases handle EA articles and their subsequent impact. The treatment of online publishing time by different publishers varied greatly [43–44]. The existence of different dates associated with the publication, such as online date, journal publication month, database index date, first tweet mentioning date, and Altmetric publication and first-seen dates, have caused confusion in the publication date of an article [43]. There were black box problems in the search of publication time in Web of Science and Scopus [8–9]. The indexing method of Scopus database for online-first article led to “citation infanticide” [10–11]. Different or incorrect indexing policies between the Web of Science and Scopus databases may lead to differences in the number of articles in the same journal [12].

Faced with the characteristics and influence of EA articles, some scholars have also discussed the appropriate time parameters for database indexing and bibliometric indication calculation. Tort et al. [36] suggested that databases use online publication dates for indexing article and calculating citation metrics. Heneberg [38] proposed that the immediacy index should be calculated based on the date of first publication (online or print, whichever comes first). Echeverría et al. [44] pointed out that when calculating bibliometric indicators of journals, the Advance Online Publication (AOP) date of articles needs to be transparent and standardized. Hu et al. [45] suggested that using online years may be more appropriate than publication years for the high citation papers (HCP) in ESI. Liu et al. [12] recommended that both Scopus and WoS CC use official publication dates consistently for indexing.

### 2.2 Comparative Studies of different bibliographic databases

Due to the continuous development and changes in the types and functions of bibliographic databases, there have been a large number of studies on database evaluation with a wide range of topics. Since the 21st century, this research field has received a lot of attention. Related studies have not only assessed the journal coverage [51,53,57,72,74–77,79,80,82,83,85], subject classification [63,64,66], retrieval functions [60,63,78,83], number of publications [49,59,63,65,66,70,84,86–88], citation counts, and associated metrics of databases [46–48,50,52–56,58–63,66–69,71–73,80,81,87], but also evaluated the comprehensiveness, availability, accuracy (or error) of various fields, which are particularly country/institution/address [13–18], funding information [19–22], DOI [23–26], author ID [27–29], document type [30–34], and language [32,35].

Many of database evaluation studies were about comparative analysis between different databases, especially WoS and Scopus, the two major bibliographic databases. In order to show the latest progress of the comparative study of these two databases, the relevant literatures in the past three years are listed in Table 1.

**Table 1:** Comparative studies of WoS and Scopus in the past three years.

Reference	Database	Comparison content
Gusenbauer [75]	56 bibliographic databases including Google Scholar, Scopus, Web of Science, etc.	Disciplinary coverage
Purnell [18]	Scopus, Web of Science, Dimensions, and Microsoft Academic	Author affiliation
Asubiaro [76]	Web of Science, Scopus, EMBASE, MEDLINE, African Index Medicus, and African Journals Online	Journal coverage
Asubiaro and Onaolapo [77]	Web of Science, Scopus, and CrossRef	Journal coverage
Kokol [22]	Scopus and Web of Science	Funding information coverage
Singh et al. [78]	Web of Science、Scopus and Dimensions	Topic retrieval quality
Asubiaro et al. [79]	Web of Science and Scopus	Journal coverage
Chinchilla- Rodríguez et al. [29]	Web of Science and Scopus	Corresponding authorship field
Gerasimov et al. [80]	Google Scholar, Web of Science, Scopus, Crossref, and DataCite	Citation coverage of datasets
Moustafa [81]	CrossRef, Web of Science, Publisher records, Google Scholar, PubMed, and Scopus	Citations
Ortega and Delgado-Quirós [82]	Dimensions, OpenAlex, PubMed, Scilit, Scopus, The Lens, and Web of Science	Coverage and overlap of retracted literature
Zhu et al. [34]	Web of Science, Scopus and journals’ websites	Document type

Ei Compendex is widely regarded as the top-notch platform for engineering literature. The comparative on it mainly involved coverage, research areas, and retrieval function (Relevant studies are shown in Table 2).

**Table 2:** Comparative studies related to Ei Compendex.

Reference	Database	Comparison content
Bartol and Hocevar [83]	Web of Science, Biosis Previews, CAB Abstracts, Chemical Abstracts, Compendex/Inspec, Francis, Medline, Pascal, and Sociological Abstracts	City-data collection and retrieval accuracy
Kostoff [84]	Science Citation Index (SCI), INSPEC, and Ei Compendex	Scientific and technological outputs
Meier and Conkling [85]	Google Scholar and Compendex	Coverage of engineering literature
Cusker [86]	Google Scholar and Elsevier Compendex	Retrieval capabilities
Cole et al. [87]	Compendex, Scopus, and Google Scholar	Citation discoverability
McAllister and Torres [88]	Web of Science and Ei Compendex	OA publications

## 3 Research question

In view of the different indexing methods of EA articles in different literature databases and their potential impact on the publication stage field, this study will take IEEE journals as an example, analyze and compare the differences of publication stage of EA articles in three typical bibliographic databases, including Web of Science Core Collection (WoS CC), Scopus and Engineering Village compendex (EI). The specific research questions include:

1. Differences in publication stages of the same EA article in WoS CC, Scopus and EI.
2. Differences in publication stage modes of different EA articles from the same journal in WoS CC, Scopus and EI.

## 4 Methodology

### 4.1 Selection of journals

IEEE (Institute of Electrical and Electronics Engineers) is one of the world's famous professional societies. Combined with the author's previous research on EA articles and their publishers (journals) in WoS CC [89], although IEEE journals do not contain a large number of EA articles, they have a certain scale, ranking 9th in 2018–2022. Therefore, considering the academic status of IEEE journals and their number of EA articles (appropriate amount of data analysis work), the author decided to choose IEEE journals as case for this study. Regarding the database, the author mainly considers whether it includes IEEE journals and its international popularity. In addition to the two comprehensive bibliographic databases (WoS CC and Scopus), the most famous engineering database (EI) in the world is also used in this study.

In order to meet the research purposes of this paper, the selected journal must meet three conditions: (1) It must be included in WoS CC, Scopus, and EI databases simultaneously; (2) It must provide an EA publication policy; (3) It must be indexed all by the EA indexing method in the above three databases. The selection process was as follows: First, in WoS CC, the search strategy of [Publisher = IEEE and Document Type = (Article OR Review)] was adopted to obtain 301 publication titles. Next, these publication titles were searched one by one on the IEEE Xplore platform. Publication titles that served as conference proceedings, did not have EA publication policies, or could not be found in IEEE were delete. The WoS database started indexing EA content in December 2017, so publication titles without publication records from 2017 and onwards were also deleted. The number of eligible journals was reduced to 186. To meet condition (3), these 186 journals were further screened to ensure that each journal contained at least one “EA record,” resulting in a list of 49 journals. “EA record” here refer to the record indexed by the WoS CC database using EA indexing method, identified by the “Early Access” tag in the Document Type field or the “Early Access Date” field [90]. These 49 journals were then checked in the Scopus and EI, and 2 of them were not found. Finally, 47 journals were identified. Of these 47 journals, only one had a unique situation, having only EA records in WoS CC and only official publication records in Scopus and EI during the period of data collection for this study. The author made a special check on this journal. According to the search results before and after the data collection time in this paper, this journal had EA records in Scopus and EI, which met the condition (3). Therefore, through the above screening process, this paper has obtained the most IEEE journals that meet the research requirements.

Here, this study used WoS CC for journal selection due to the different EA indexing rules of the three databases. Specifically, the WoS CC database retains indexing information for both early access records and official publication records, while Scopus and EI delete early access records after indexing official publication records. If a journal's early access records in Scopus and EI are deleted during data retrieval, it will not be possible to determine whether this journal meet the requirements for this study. As with the special case above, this paper has avoided this problem by using WoS CC. If a journal has early access records in Scopus and EI, but only official publication records in WoS CC, this means that WoS CC's indexing rules for this journal are still traditional rules, rather than EA indexing rules [does not meet condition (3)], so using WoS CC has avoided some unnecessary workload.

### 4.2 Creation of data sets

Records of the above 47 journals published in 2021–2023 were searched in the three databases. For the same journal, the search was completed on the same day. The total records of 43,011 (WoS CC), 45,212 (Scopus), and 44,937 (EI) were collected from 20 June to 2 July 2023. The records for individual journals ranged from 87 to 4340 (WoS CC), 83–4531 (Scopus), and 83 to 4489 (EI).

In this study, all records from three databases were pair-to-pair compared. The DOI served as the criterion for determining whether a record was the same. This paper classified records into three types of data sets based on the number of databases in which they were located: records with unique DOI in one database (Type 1), records with the same DOI in two databases (Type 2), and records with the same DOI in three databases (Type 3). The meanings of these three types of records were as follows: records that appeared in only one database, records that appeared in two databases, and records that appeared in three databases. According to the different databases, these three types of data sets were further divided into 7 sub-data sets (see Table 3).

**Table 3:** Information of data sets and publication stage modes.

Type of data set	DOI matching	Database	Sub-data set	Publication stage mode	Label of publication stage mode	Whether there is a difference in publication stages
Type 1: Records with a unique DOI in one database	DOI_W_ ≠ DOI_S_ ≠ DOI_E_	WoS CC	D1	PS_W_ = EA	PS1	No
				PS_W_ = OP	PS2	No
		Scopus	D2	PS_S_ = EA	PS3	No
				PS_S_ = OP	PS4	No
		EI	D3	PS_E_ = EA	PS5	No
				PS_E_ = OP	PS6	No
Type 2: Records with the same DOI in two databases	DOI_W_ = DOI_S_ ≠ DOI_E_	WoS CC and Scopus	D4	PS_W_ = PS_S_ = EA	PS7	No
				PS_W_ = PS_S_ = OP	PS8	No
				PS_W_ = EA, PS_S_ = OP	PS9	Yes
				PS_W_ = OP, PS_S_ = EA	PS10	Yes
	DOI_W_ = DOI_E_ ≠ DOI_S_	WoS CC and EI	D5	PS_W_ = PS_E_ = EA	PS11	No
				PS_W_ = PS_E_ = OP	PS12	No
				PS_W_ = EA, PS_E_ = OP	PS13	Yes
				PS_W_ = OP, PS_E_ = EA	PS14	Yes
	DOI_S_ = DOI_E_ ≠ DOI_W_	Scopus and EI	D6	PS_S_ = PS_E_ = EA	PS15	No
				PS_S_ = PS_E_ = OP	PS16	No
				PS_S_ = EA, PS_E_ = OP	PS17	Yes
				PS_S_ = OP, PS_E_ = EA	PS18	Yes
Type 3: Records with the same DOI in three databases	DOI_W_ = DOI_S_ = DOI_E_	WoS CC, Scopus and EI	D7	PS_W_ = PS_S_ = PS_E_ = EA	PS19	No
				PS_W_ = PS_S_ = PS_E_ = OP	PS20	No
				PS_W_ = PS_S_ = EA, PS_E_ = OP	PS21	Yes
				PS_W_ = PS_E_ = EA, PS_S_ = OP	PS22	Yes
				PS_S_ = PS_E_ = EA, PS_W_ = OP	PS23	Yes
				PS_W_ = PS_S_ = OP, PS_E_ = EA	PS24	Yes
				PS_W_ = PS_E_ = OP, PS_S_ = EA	PS25	Yes
				PS_S_ = PS_E_ = OP, PS_W_ = EA	PS26	Yes

Note: W, S, and E in the DOI subscript in the table represented WoS CC, Scopus, and EI, respectively.

According to some previous studies, there may be omissions and errors in DOI in bibliographic databases [23–26]. In this study, the problem of DOI missing was also found in the above comparison. There were 48 DOI missing records in WoS CC (0.11% of the total records 43011), 7 in EI (0.02% of the total records 44937), and none in Scopus. There were several possible cases of missing DOI. (1) The record existed in only one database, and the DOI was missing; (2) The record was missing the DOI in one of the databases, but had the same DOI in the other two databases; (3) The same record had two indexed records in a database, one of which was an EA record without a DOI (belonging to the DOI missing record), and the other was an official publication record with a DOI. Due to the low proportion of missing DOI records, this paper directly classified them into the Type 1 data set for analysis, and did not do further processing (such as using the title to check, etc.). For possible DOI errors, such as the DOI of a particular article in different databases may be different (not a problem of missing DOI), this paper did not make special comparison and confirmation.

### 4.3 Qualitative analysis of publication stage modes

Although EA records in the three databases have different field markers, they have the same two basic Publication Stages (PS), namely Early Access (EA) and Official Publication (OP). Combining the three databases in this study, there were six publication stages: PS_W_ = EA, PS_S_ = EA, PS_E_ = EA, PS_W_ = OP, PS_S_ = OP, PS_E_ = OP. Here, W, S, and E in the PS subscript represented WoS CC, Scopus, and EI, respectively.

The seven sub-data sets above had a total of 26 possible combinations of publication stages, which were referred to as “publication stage modes” in this paper. These modes were labeled separately in Table 3. Among these 26 modes, 14 were defined as the “undifferentiated publication stage modes”, including publication stages of unique records and those of the same record that did not differ across different databases. This type of modes covered 6 modes (PS1-PS6) in D1-D3 and 8 modes (PS7, PS8, PS11, PS12, PS15, PS16, PS19, PS20) in D4-D7. The remaining 12 modes (PS9, PS10, PS13, PS14, PS17, PS18, PS21-PS26) were defined as “differentiated publication stage modes”, meaning that publication stages of the same record varied across different databases.

### 4.4 Quantitative analysis of publication stage modes

Based on the above data sets and publication stage modes, this paper quantitatively analyzed the Record Numbers (*RN*), Record Proportion (*RP*), and their average values of various data sets and publication stage modes. To analyze the distribution of journals, this study identified journals whose *RN* ≠ 0 and counts the corresponding Journal Numbers (*JN*). The specific process and calculation equation was as follows.

a. The *RN* of each journal in the 7 sub-data sets (*RN*_*i,j*_) was counted. Equation 1 was used to calculate the average value of *RN*_*i,j*_ of a certain sub-data set ($\overset{―}{{\text{RN}}_{\text{j}}}$), and equations 2 and 3 were used to calculate the *RP* of each journal (*RP*_*i,j*_) and its average value ($\overset{―}{{\text{RP}}_{\text{j}}}$).


$$\overset{―}{RN_{j}}=\frac{\sum_{i=1}^{47}RN_{i,j}}{47}$$ (1)

$$RP_{i,j}=\frac{RN_{i,j}}{\sum_{j=1}^{7}RN_{i,j}}\times 100\;\%$$ (2)

$$\overset{―}{RP_{j}}=\frac{\sum_{i=1}^{47}RP_{i,j}}{47}$$ (3)

b. The *RN* of the 26 publication stage modes of each journal (*RN*_*i*_*,*_*k*_) was counted. Equation 4 was used to calculate the average value of *RN*_*i*_*,*_*k*_ of a certain publication stage mode ($\overset{―}{{\text{RN}}_{\text{k}}}$). Equation 5 was used to calculate the *RP* of a certain publication stage mode in its sub-data set (*RP*_*i,k*_), and equation 6 was used to calculate the average value of *RP*_*i,k*_ ($\overset{―}{{\text{RP}}_{\text{k}}}$).


$$\overset{―}{RN_{k}}=\frac{\sum_{i=1}^{47}RN_{i,k}}{47}$$ (4)

$$RP_{i,k}=\frac{RN_{i,k}}{\sum_{k=k\min}^{k\max}RN_{i,k}}\times 100\;\%$$ (5)

$$\overset{―}{RP_{k}}=\frac{\sum_{i=1}^{47}RP_{i,k}}{47}$$ (6)

In equations 1–6, *i* was the *i* journal (a total of 47 journals), *j* was the *j* sub-data set (a total of 7 sub-data sets), and *k* was the *k* publication stage mode (a total of 26 publication stage modes). The value range of *k* in equation 5 was divided into 1–2, 3–4, 5–6, 7–10, 11–14, 15–18, and 19–26 according to the sub-data set and its corresponding publication stage modes.

c To analyze the distribution of journals in each sub-data set, journals with *RN*_*i,j *_≠ 0 were identified, and the Journal Numbers (*JN*_*j*_) were counted. To investigate the distribution of journals in each publication stage mode, journals with *RN*_*i*_*,*_*k *_≠ 0 were identified, and the Journal Numbers (*JN*_*k*_) were counted.
d To analyze and visually display the overall situation of the *RP*_*i,j*_ and the distribution of journals in each sub-data set, this paper drew figures of the *RP*_*i,j*_ in three types of data sets (Type 1, Type 2, and Type 3) respectively, and made a comparison.
e To analyze and visually display the *RP*_*i,k*_ and the journals distribution of publication stage modes contained in each sub-data set, this paper drew figures of the *RP*_*i,k*_ of publication stage modes contained in each sub-data set, respectively, and made a comparison.
f Based on Table 3, the calculation results of equation 2 and equation 5, and the statistical results of step c, this paper counted the number of sub-data sets, the number of publication stage modes, and the number of differentiated publication stage modes contained in each journal, and displayed with figures.

### 4.5 Analysis of research questions

For research question 1, the main contents of the analysis include: Is there a difference in the publication stage of the same EA article in different databases? If there are differences, what is the publication stage mode? What is the quantity and proportion? The general analysis idea is: First determine what type of data set that the record belongs to. If it is Type 1, it means that there is no so-called difference in the publication stage, as shown in the six publication stage modes listed in Table 3 (PS1-PS6). If it is Type 2 and Type 3, then further analyze the proportion of PS7-PS26 listed in Table 3. There are 8 “undifferentiated publication stage modes”, including PS7, PS8, PS11, PS12, PS15, PS16, PS19, PS20, and 12 “differentiated publication stage modes”, including PS9, PS10, PS13, PS14, PS17, PS18, PS21-PS26. If the number or proportion of these 12 differentiated modes is not 0, it means that the publication stage of the same record is different in different databases. Through the specific number and proportion information, we can know whether these 12 modes exist, which modes are the main ones. And through the number of journal distributions, we can also know whether such differences are common and whether it has certain rules.

For research question 2, the main contents of the analysis include: Is there a difference in the publication stage of different EA articles from the same journal in different databases? If there are differences, how many publishing stage modes are there? What is it exactly? What is the quantity and proportion? The analysis can be divided into three steps. The first step is to analyze the record number, proportion and average value of each data set in each journal. The second step is to analyze the record number, proportion and average value of each publication stage mode in each journal. The third step, based on the results of the first and second steps, analyzes how many types of data set that each journal includes, how many publication stage modes involved and how many “differentiated publication stage modes” involved. If more than one data set type or publication stage mode is involved in a journal, it can indicate that the publication stage mode of different EA articles in the journal is different. Based on the above results, we can also obtain the journal distribution number of each data set, the journal distribution number of each publication stage mode, and the relationship between the journal distribution number and the number of data sets, the number of publication stage modes, and the number of “differentiated publication stage modes”. Through the above journal distribution information, we can explain whether the difference is universal. At the same time, we can analyze whether the difference among different journals has certain regularity by combining the specific information of quantity and proportion.

## 5 Results and discussions

### 5.1 Research question 1- Differences in the publication stage of the same EA article

By analyzing the database where records were located, it was found that the records from the 47 IEEE journals involve all seven sub-data sets of three types (D1-D7). The number of records in descending order was D7 (WoS CC, Scopus, and EI) ˃ D6 (Scopus and EI) ˃ D1 (WoS CC) ˃ D2 (Scopus) ˃ D3 (EI) ˃ D5 (WoS CC and EI) ˃ D4 (WoS CC and Scopus). Among them, D7 accounted for the largest proportion, reaching 89.96% (see Table 4).

**Table 4:** The *RN*, *RP*, *JN* of D1-D7 and PS1-PS26.

Sub-data set	$\sum {RN}_{i,j}$	$\overset{―}{{\text{RN}}_{\text{j}}}$	$\overset{―}{{\text{RP}}_{\text{j}}}$ (%)	*JN*_*j*_	Publication stage mode	$\sum {\text{RN}}_{\text{i,k}}$	$\overset{―}{{\text{RN}}_{\text{k}}}$	$\overset{―}{{\text{RP}}_{\text{k}}}$(%)	*JN*_*k*_
D1	743	16	3.06	42	PS1	157	3	18.44	20
					PS2	586	12	70.92	39
D2	349	7	0.71	34	PS3	229	5	62.27	32
					PS4	120	3	10.07	7
D3	67	1	0.14	5	PS5	13	0	7.56	5
					PS6	54	1	3.08	2
D4	41	1	0.19	13	PS7	1	0	0.71	1
					PS8	38	1	24.11	12
					PS9	2	0	2.84	2
					PS10	0	0	0.00	0
D5	51	1	0.21	9	PS11	16	0	7.45	4
					PS12	33	1	8.87	5
					PS13	0	0	0.00	0
					PS14	2	0	2.84	2
D6	2646	56	5.73	47	PS15	1906	41	63.35	44
					PS16	691	15	35.65	39
					PS17	20	0	0.41	1
					PS18	29	1	0.59	1
D7	42176	897	89.96	47	PS19	4564	97	10.04	40
					PS20	35149	748	85.10	47
					PS21	241	5	0.15	1
					PS22	69	1	0.04	1
					PS23	224	5	0.68	16
					PS24	187	4	0.12	2
					PS25	210	4	0.31	20
					PS26	1532	33	3.55	33

Note: *RN*, *RP*, *JN* in the table represented Record Numbers, Record Proportion, and Journal Numbers, respectively. In the subscript, *i* was the *i* journal (a total of 47 journals), *j* was the *j* sub-data set (a total of 7 sub-data sets), and *k* was the *k* publication stage mode (a total of 26 publication stage modes).

**Fig 1:**
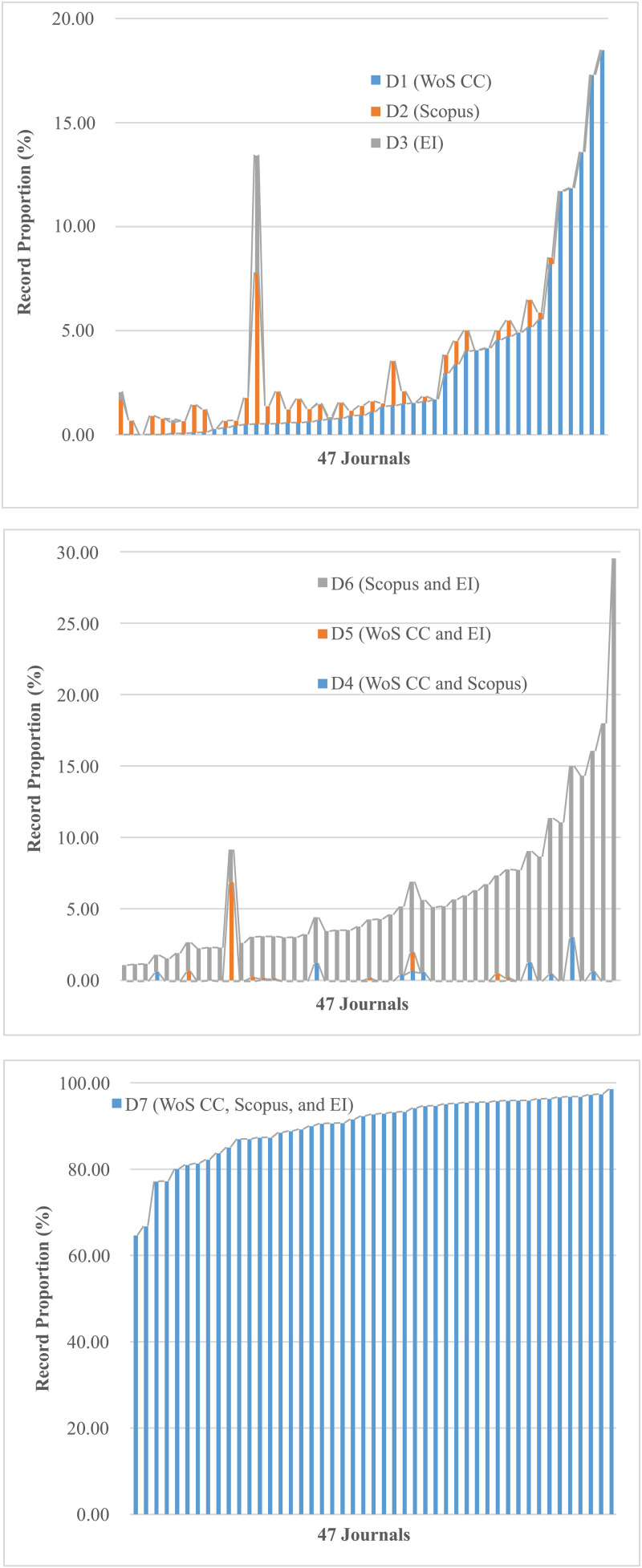
The record proportion of data sets (D1-D7).

In the Type 1 data set (D1, D2, and D3), records existed in only one database and there was no difference in their publication stages. Both the number (*RN*) and proportion (*RP*) of such records were low (as shown in Table 4 and Fig 1). The average proportion of D1 (WoS CC) records ($\overset{―}{{\text{RP}}_{\text{j}}}$) was 3.06%, and $\overset{―}{{\text{RP}}_{\text{j}}}$ of D2 (Scopus) and D3 (EI) records were both less than 1.00%.

In the Type 2 data set (D4, D5, and D6), records existed in two databases simultaneously. As could be seen from Table 4, the absolute number of D4 (WoS CC and Scopus) and D5 (WoS CC and EI) that differed in the publication stage of PS9 (PS_W_ = EA, PS_S_ = OP) and PS14 (PS_W_ = OP, PS_E_ = EA) was relatively small (only 4 records), while the absolute number of D6 (Scopus and EI) that differed in the publication stage of PS17 (PS_S_ = EA, PS_E_ = OP) and PS18 (PS_S_ = OP, PS_E_ = EA) was relatively large (49 records in total). In D4 (WoS CC and Scopus), PS8 (PS_W_ = PS_S_ = OP) was the main publication stage mode, PS10 (PS_W_ = OP, PS_S_ = EA) had no records, while PS7 (PS_W_ = PS_S_ = EA) and PS9 (PS_W_ = EA, PS_S_ = OP) only had 1 and 2 records, respectively. In D5 (WoS CC and EI), PS11 (PS_W_ = PS_E_ = EA) and PS12 (PS_W_ = PS_E_ = OP) were the main publication stage modes. PS13 (PS_W_ = EA, PS_E_ = OP) had no records, while PS14 (PS_W_ = OP, PS_E_ = EA) only had 2 records. In D6 (Scopus and EI), PS15 (PS_S_ = PS_E_ = EA) and PS16 (PS_S_ = PS_E_ = OP) were the main publication stage modes. PS17 (PS_S_ = EA, PS_E_ = OP) and PS18 (PS_S_ = OP, PS_E_ = EA) had 20 and 29 records respectively, but they all came from only one journal.

In the Type 3 data set (D7), records existed in three databases simultaneously. Table 4 showed that the total number of records ($\sum {\text{RN}}_{\text{i,k}}$) of PS20 (PS_W_ = PS_S_ = PS_E_ = OP) was 35149, ranking first among all publication stage modes, with an average proportion of 85.10%, significantly higher than other models. The PS19 (PS_W_ = PS_S_ = PS_E_ = EA) ranked second with the record number of 4564. The absolute number of records with “differentiated publication stage models” (PS21-PS26) is large (2463 in total), and the proportion of PS26 (PS_S_ = PS_E_ = OP, PS_W_ = EA) was the highest (3.55%), while the proportion of the other 5 modes was less than 1.00%.

In summary, the results showed that the publication stage of the same EA article in IEEE journals is different in different databases. In the above 12 “differentiated publication stage modes”, in addition to PS10 (PS_W_ = OP, PS_S_ = EA) and PS13 (PS_W_ = EA, PS_E_ = OP), the remaining 10 modes all appeared, of which PS26 (PS_S_ = PS_E_ = OP, PS_W_ = EA) was the most common. The relative number of records (proportion) of each “differentiated publication stage model” was mostly below 1.00%, which is “uncommon”. But judging by the absolute number of records (2516 in total), the discrepancy is “common”, which is remarkable.

If the publication stage of an article is inconsistent in different databases, it may bring about multiple problems, including research performance standards, calculation of measurement indicators, and setting of retrieval strategies, etc.

Different research institutions and evaluation departments may have different standards for the certification of the publication time of articles (online access time or official publication time). Then which database to take as the criterion will become a problem, because different databases are likely to lead to different results. When an article is indexed by multiple databases, there is a certain risk of relying solely on the search results from a single database. Therefore, this point needs to be taken into consideration in the formulation of performance evaluation policies. It is recommended to verify multiple databases or refer to the journal website (if the journal website is updated in a timely manner), which may further increase time and price costs.

When counting the number of papers and their corresponding citations and other related indicates, the time data associated with the publication stage may become a cause of result deviation, especially when the online access time and official publication time are not in the same year. Correspondingly, this will also affect the setting of retrieval strategies, and the impact of different time search fields on results should be considered.

### 5.2 Research question 2 – Differences in publication stage modes of different EA articles from the same journal

Firstly, from the perspective of sub-data sets. For the same journal, it is also necessary to start from the sub-data set to analyze whether it contains different publication stage modes. According to the journal distribution results of seven data sets (see Table 4), the ranking of the number of journals was as follows: D7 (WoS CC, Scopus, and EI) =D6 (Scopus and EI) ˃ D1 (WoS CC) ˃ D2 (Scopus) ˃ D4 (WoS CC and Scopus) ˃ D5 (WoS CC and EI) ˃ D3 (EI).

**Fig 2:**
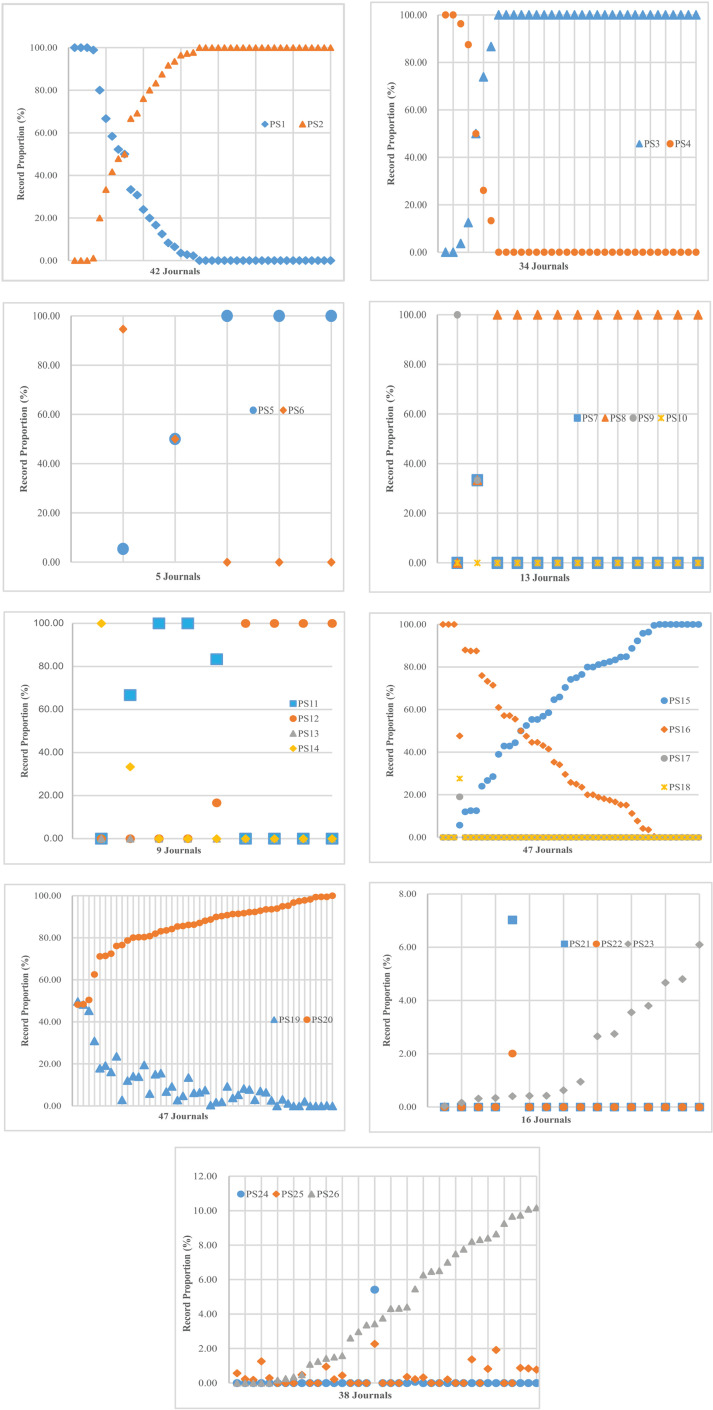
The record proportion of publication stage modes (PS1-PS26).

Secondly, from the perspective of publication stage modes. According to Table 4, regarding the “undifferentiated publication stage modes” in a single database, the journal distribution numbers of PS1 (PS_W_ = EA), PS2 (PS_W_ = OP), and PS3 (PS_S_ = EA) is relatively large, which are 20, 39 and 32 respectively. In the two databases, the journal distribution numbers of PS15 (PS_S_ = PS_E_ = EA), PS16 (PS_S_ = PS_E_ = OP) and PS8 (PS_W_ = PS_S_ = OP) reached 44, 39 and 12 respectively, and the journal numbers of other modes were less than or equal to 5. In the three databases, the number of journals in PS19 (PS_W_ = PS_S_ = PS_E_ = EA) and PS20 (PS_W_ = PS_S_ = PS_E_ = OP) was 40 and 47 respectively. Regarding the “differentiated publication stage modes”, in the two databases, PS9 (PS_W_ = EA, PS_S_ = OP) and PS14 (PS_W_ = OP, PS_E_ = EA) existed in only two journals, with only one record per journal. P17 (PS_S_ = EA, PS_E_ = OP) and P18 (PS_S_ = OP, PS_E_ = EA) appeared in only one journal, with 20 and 29 records respectively. PS24 (PS_W_ = PS_S_ = OP, PS_E_ = EA) existed in only 2 journals, with 186 and 1 record, respectively. In the three databases, PS21 (PS_W_ = PS_S_ = EA,PS_E_ = OP), PS22 (PS_W_ = PS_E_ = EA, PS_S_ = OP), and PS23 (PS_S_ = PS_E_ = EA, PS_W_ = OP) had the EA stage in two databases and the OP stage in one database. PS21 and PS22 appeared in only one journal, while PS23 appeared in 16 journals. PS24 (PS_W_ = PS_S_ = OP, PS_E_ = EA), PS25 (PS_W_ = PS_E_ = OP, PS_S_ = EA), PS26 (PS_S_ = PS_E_ = OP, PS_W_ = EA) had the OP stage in two databases and the EA stage in one database. PS24 appeared in only 2 journals, while P25 and P26 involved a larger number of journals, 20 and 33 respectively. Looking further at Fig 2, different publication stage modes showed their own characteristics in the record proportion and journal distribution, and it seemed to be no rule to follow.

**Fig 3:**
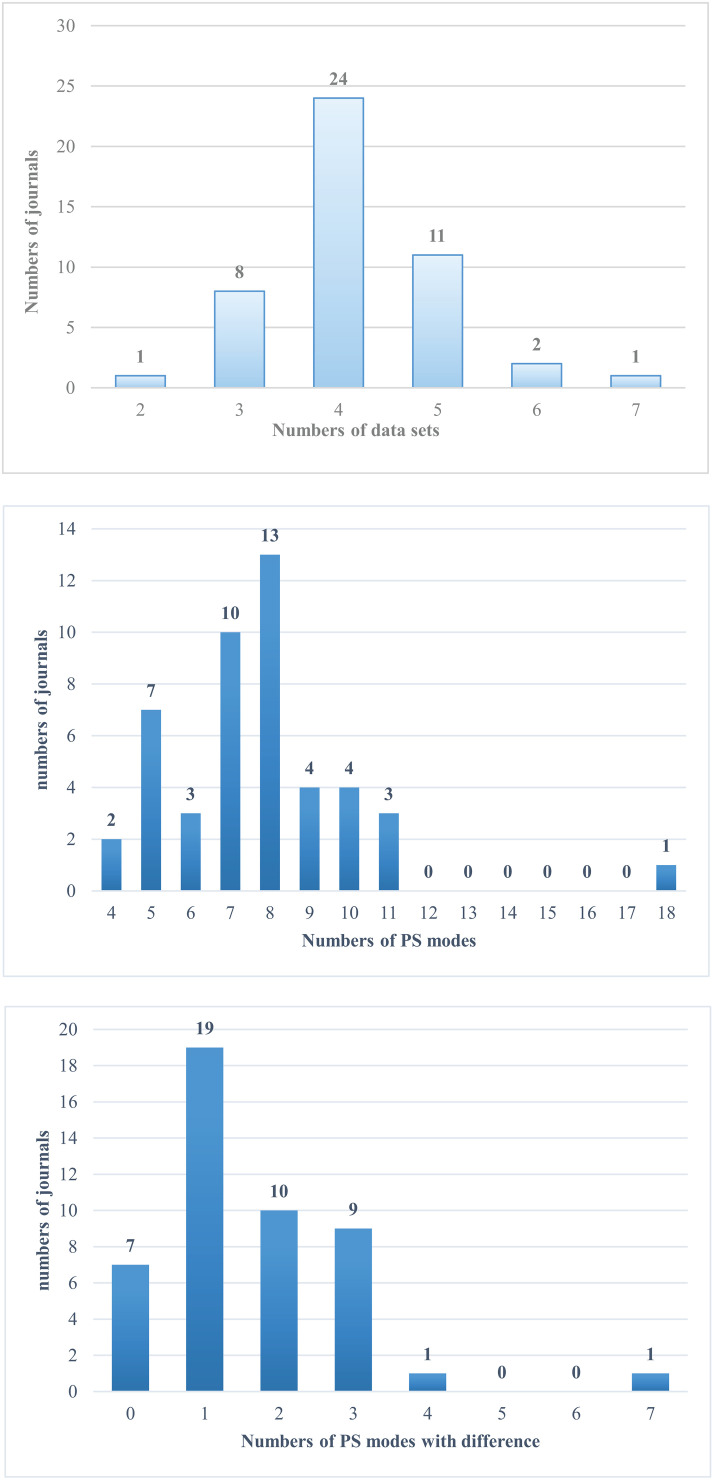
Relationship between numbers of journal with numbers of sub-data sets, publication stage modes and differentiated publication stage modes.

Finally, according to the summary results in Fig 3, among 47 IEEE journals, each journal had 2 or more different sub-data sets, of which 24 journals (accounting for 51.06%) had 4 ones at the same time, 11 journals had 5 ones, 8 journals had 3 ones, 2 journals had 2 ones, and one journal even had 7 ones at the same time. In addition, each of the 47 journals contained at least 4 publication stage modes simultaneously, of which 13 journals contained 8 modes, 10 journals contained 7 modes, 7 journals contained 5 modes, 4 journals contained 9 modes, 4 journals contained 10 modes, and 3 journals contained 6 modes, 3 journals contained 11 modes, and one journal even contained 18 modes simultaneously. Among the 47 journals, except for 7 journals that did not had “undifferentiated publication stage mode”, the remaining 40 journals (85.11%) all had one or more differentiated modes, among which 19 journals had one differentiated mode, 10 journals had 2 differentiated modes, and 9 journals had 3 differentiated modes, one journal had 4 differentiated models, and one journal had 7 differentiated models.

Based on the above results, it could be concluded that the publication stage modes of different EA articles in the same IEEE journal were different, and the publication stage modes contained in the same IEEE journal were diverse and complex. At the same time, this phenomenon not only appeared in a single journal, with a certain universality, but also seemed to have no special rules to follow, with uncertainty.

This result undoubtedly indicates that it is difficult to examine a certain journal using a fixed publication stage model. To comprehensively understand the inclusion and publication status of articles of a certain journal in different databases, specific analysis is required on a case-by-case basis. Generally, databases index bases on the original data provided by journals. However, the diversity presented in results of this study would remind databases and publishers to think about and improve the indexing process and rules, because both may lead to such results due to their own reasons. Specifically, possible reasons include different original data provided by publishers to databases, different indexing methods and efficiencies of databases, errors in the original data, and errors of indexing, etc. In addition, it was found in the results that some differentiated modes only occurred in one or two journals. If this is an accidental situation, perhaps it can be effectively avoided through regular checks by the database and journal publishers.

## 6 Conclusion and limitation

This exploratory study revealed that in the three typical bibliographic databases (WoS CC, Scopus and EI), the publication stage of the same EA article in IEEE journals was different, and the publication stage mode of different EA articles in one IEEE journal was also different. In IEEE journals, these differences were universal, complex and uncertain. It is hoped that the results of this study will draw people's attention to the differences in the EA articles indexing of different bibliographic databases. Therefore, it is unreliable to use a single database to determine the publication stage and the corresponding publication time information of a certain article or a certain journal. It is hoped that results of this study can draw the attention of various stakeholders, including researchers, research institutions and evaluation departments, as well as databases and journal publishers, to the indexing differences of EA articles across different bibliographic databases. This paper had some limitations in the scope of the study. If the future study is carried out with a wider range of journals and more EA article samples, the comprehensiveness of the study results will be further improved.

## Supporting information

S1 File Data of Table 4. (XLSX)

S2 File Data of Fig 1. (XLSX)

S3 File Data of Fig 2-PS1-PS6. (XLSX)

S4 File Data of Fig 2-PS7-PS18. (XLSX)

S5 File Data of Fig 2-PS19-PS26. (XLSX)

S6 File Data of Fig 3. (XLSX)
